# "The Hospital" Nursing Mirror

**Published:** 1896-08-08

**Authors:** 


### "The Hospital" Nursing Mirror.

Being the Extra Nursing Supplement of "The Hospital" Newspaper.

[Contributions for this Supplement should be addressed to the Editor, The Hospital, 428, Strand, London, W.C., and should have the word "Nursing" plainly written in left-hand top corner of the envelope.]

### News from the Nursing World.

### A GENEROUS GIFT.

We are glad to see that £500 has been given to the Paddington Green Children's Hospital by a gentleman in memory of his daughter. The money is to be spent in furnishing and decorating a new ward in the hospital. The mural decorations promise to be very charming. They are to take the form of pictures representing Æsop's Fables, executed in tiles—a cleanly and durable way of ornamenting to which few can object.

### NURSING QUARTERS AT SHOOTER'S HILL.

At the magnificent new hospital called the Brook Hospital, just erected by the Metropolitan Asylums Board, great attention has evidently been paid to making the nurses really comfortable. The sleeping arrangements are good, bright, and airy, whilst the sitting and dining rooms are some of the pleasantest apartments we have seen for a long time. The views from the windows are beautiful, and the nurses' home is quite apart from the scene of work. The matron's quarters are situated in the domestic block, and are very complete and pleasant also. There is a separate entrance, so that visitors can be admitted without running the risk of infection, and a nice little dining, sitting, bedroom, bath-room, and pantry completes the suite of apartments. The Brook Hospital is more like a small town than one institution, and the post of matron will be a most responsible one, and one that will demand an immense amount of energy and administrative capacity. There is a suggestion that Miss Bann should have an assistant superintendent of nurses under her, and we cannot help thinking this help will be found necessary if the matron's strength and capacity is to be preserved. Miss Bann is one of those bright, willing, and energetic people who need protecting from themselves. We congratulate the Metropolitan Asylums Board on the choice they have made of an officer to fill this important post, and prophesy that fever nursing at Shooter's Hill will be a much sought for experience amongst nurses.

### INTERFERING RELATIVES.

It has become quite a fashionable occupation to take up the cause of the oppressed and cruelly treated nurse. The difficulty of finding nurses to act as *bonâ fide* object-lessons of the alleged cruelty and oppression is, however, becoming greater day by day. The genuine working nurse repudiates the position of an overburdened and underfed victim which she is asked to assume; and even the inexperienced and often unreasonable probationer sometimes abandons the magnification of petty grievances for the more healthy and happier occupation of taking a genuine interest in her work for others. A case in point has just taken place at the Lewisham Infirmary, where the authorities have been attacked in a most indignant manner by the father and uncle of a probationer for subjecting her to "objectionable and menial duties," and to "the insults of a fellow nurse." On an inquiry being held, the probationer failed to substantiate any of the complaints made for her, and asked to be allowed to remain on the infirmary staff. The authorities, under the circumstances, have asked the complaining father to apologise. What a pity it is that relatives are not more judicious and practical.

### DOCTORS, NURSES, AND SUNLIGHT SOAP.

Brighton has just held its Hospital Saturday collection, and in order to add to the attractions of the occasion, the friendly and trade societies threw in their lot with the Hospital Saturday Committee. Besides the usual pageants, which bands, banners, and costumes of the various associations represent, tableaux on wheels were provided. Thus the spectacle of illness was paraded through the streets, represented by a hospital nurse beside a small patient in a cot, and by a "living picture" giving a celebrated and pathetic study, "The Doctor." In company with these came "Sunlight Soap"! Surely the subject of illness is not one for curiosity and display, and we think such masquerading adds greatly to the objectionable features of street collections, and is not likely to heighten respect for the healing art, when doctors, patients, nurses, sorrowing friends, and Sunlight Soap are all jumbled up together.

### EIGHT HOURS FOR NURSES.

A champion of nurses who has lately arisen in the columns of an Irish newspaper suggests that the nursing staff of hospitals should be so increased as to admit of an eight hours working day for all nurses—divided in two divisions of four hours each. Such an arrangement might delight the heart of a nurse who had taken up nursing purely as a means of livelihood and had no further interest in the care of the sick; but to an energetic nurse, wholly devoted to her work, an arbitrary limitation of the kind could only be regarded as irksome and tyrannical. In the matter of long hours in many institutions nurses still have just cause for complaint, but public opinion, the enlightenment of hospital administrators, and the exertions of the friends of nurses is gradually fixing a reasonable standard, whereby the hours off duty are more frequent and longer, to the benefit of all concerned. The eight hour arrangement is not the unmitigated success with the working classes to warrant a very strong recommendation of its claims to nurses.

### AN INVALID KITCHEN.

In connection with the Southport and Birkdale District Nursing Society an invalid kitchen has been established since October last. No less than 5,000 meals have been provided for poor patients in their own homes since then. The greatest care is taken that the assistance should be worthily bestowed, and all the provisions are paid for by the friends of the patients. The meals are distributed by a volunteer band of lady helpers. They are conveyed in nice little carriers, divided into compartments. They are enveloped in green covers, which keep the food beautifully hot. The tramway companies collect the carriers and deposit them at a central depôt, free of charge, which is a most useful and kindly act on their part. We are sorry to learn that the kitchen is carried on at a loss, and should like to hear that some kind friend has endowed so useful an institution with sufficient means to cover working expenses until it is self-supporting, which should be quite possible under good management, and with its benefits better known throughout Southport.

### NURSES FOR SUFFOLK.

A meeting was recently held at Ipswich to consider a proposal to establish a County Nursing Association for East and West Suffolk and the borough of Ipswich. The Rev. A. Peile, president of the Queen's Jubilee Institute, and Miss Sumpster, secretary of the Lincolnshire Nursing Association. In forming the new society the Rev. A. Peile heartily recommended, and Miss Sumpster explained the organisation of the Lincolnshire Association. It will be remembered that some time ago we explained this scheme fully, which is one, briefly, of giving cottage women a short training in nursing, and employing them to nurse the poor in their own homes, this plan being adopted from economic reasons, and it being stated that such a class of nurse was more satisfactory than the fully-trained nurse. The meeting agreed to establish an association on the lines of the Lincolnshire Association, and a committee was there and then formed under the presidency of Lady Bristoe, and subscriptions were announced which will start the association on a good financial basis.

### NURSING IN RIO DE JANEIRO.

Whether they join an institution abroad, or undertake nursing on their own account, we feel we cannot advise nurses too strongly to make all enquiries and the most definite arrangements before starting. Difficulties which are trivial near home are seriously inconvenient when they occur far away, and an expensive journey, or a condition sometimes of actual want, has to be faced. It appears that at Rio de Janeiro there is no definite agreement drawn up in respect to the nurses being sent out to private cases from the Stranger's Hospital. It seems to have been the custom, however, and recently a nurse objecting to fall in with the arrangement was dismissed, several others resigning in sympathy with her. Such disagreeable proceedings could well have been avoided had the nurses made definite and full enquiries beforehand.

### DISTRICT NURSING AT NEWCASTLE.

The Cathedral Nurse and Loan Society at Newcastle does a most important work, and we are sure it need not feel any grave anxiety that a slight deficiency exists in respect of funds. The society has the sympathy of the community, and we feel sure will not be allowed to remain long in need. There is an invalid kitchen in connection with the institution, and its benefits have been liberally dispensed, and thousands are the quarts of milk, besides large amounts of beef-tea, eggs, &c., which have found their way to poor patients. The society works over a large field and numerous branch districts are supplied with a nurse of nurses, for each of whom £100 a year is paid. Then the society administers two convalescent homes, and thus it will be seen that the work of the society is a really important one. At the recent annual meeting the vicar of Yarrow said, in speaking of the influence exercised by the nurses in the poor homes which they visit, that they went to all denominations alike, and the effect was very softening and was a step in the direction of the reunion of Christendom.

### NURSING AT SHEFFIELD.

The Sheffield Nurses' Home and Training Institution presents a very favourable report of work done during the past year. The nurses have earned £1,387. The home is not entirely self-supporting, but, owing to the interest which is taken in it locally, funds have been collected which have covered expenses. There is a district nursing branch in connection with the home, and the institution supplies nurses to districts which can afford to pay for their services.

### RADSTOCK NURSING SOCIETY.

A bazaar has just been held in aid of this society, under the patronage of Lord Carlingford, and as there was a large attendance we hope the society have reaped a handsome sum. Besides the usual sales at various stalls, there were many other attractions, such as maypole dances, ladies' washing competition, gentlemen's hat-trimming competition, concerts, and a cricket match, ladies against gentlemen, who played with broomsticks.

### THE BAZAAR AT LANCASTER.

We are glad to learn that the proceeds of the bazaar in aid of the Lancaster Infirmary amounted to the goodly sum of £3,814. Out of this expenses have to be paid, but, on the other hand, all the articles for sale are not yet disposed of, and many ladies have undertaked to dispose of them privately, and to hold a sale of them next year. It was a very happy idea to let various districts be responsible for their own stall, as this naturally stimulated local interest and competition.

### SHORT ITEMS.

The nurses who attended the inspection of Messrs. Bovril's manufactory last week were highly pleased with all they saw, and by the hospitable reception which they met.—The committee of the Gloucester District Nursing Society testify to the necessity and popularity of the society, and report a year of useful work and fair financial prosperity.—Two concerts in aid of the Belvoir District Nursing Association were given last week by Lady Ada Scott, at Belvoir Castle.—Ireland has not the reputation of leading in hospital movements, but the decision at the Limerick Union to extend the limit of the age of a nurse employed by them from 37 to 47, on account of the competency of a nurse of this age for a vacant post, is certainly a progressive movement.

### Hygiene: For Nurses.

By John Glaister, M.D., F.F.P.S.G., D.P.H.Camb., Professor of Forensic Medicine and Public Health, St. Mungo's College, Glasgow, &c.

### XVIII.—PERSONAL HABITS IN RELATION TO HEALTH—HEREDITARY AND ACQUIRED.

It may be said that the personal habits of a people find their reflex in the standard of national health. These habits, if objectionable, may be minimised or intensified, however, by the geographical position which the particular country occupies, and by other factors. This becomes still more true of the inhabitants of certain districts of populous places which are the habitats and resorts of the vicious, the thriftless, the lazy, and the dirty. Such places are deplorable blots on the social landscape, and no scheme of amelioration has as yet solved the problem of successfully overcoming the evils caused by them.

The cardinal point around which personal and public health revolves is cleanliness—of the person, of food, and of the home. Probably the oldest sanitary code in existence is that which was enacted during the Mosaic Dispensation, and which is carried out to this day by all faithful Jews in whatever part of the world they are situated. It would be well for hygiene if the injunctions laid down in that code could be carried out, if not in their entirety, at least in large measure, as part of the religion of all peoples. In the forefront of it stands cleanliness, individual, dietetic, and domestic; and there is no doubt that the longevity and general excellent standard of health of Jews are to be attributed to the observance of the laws laid down in the Bible and the Talmud. The plentiful use of water, the frequent bathing of the person, the cleansing and disinfection of the home, and many like important rules, are prominent injunctions of the Mosaic code.

Personal cleanliness ought not, at this time of day, to be a doctrine which needs to be inculcated; unfortunately, however, it is still one which requires teaching. The bulk of persons have much to learn regarding the physiology of the skin; when the fact is considered that millions of sweat and oil glands open on the surface of the body, it will be at once apparent that cleanliness is essential to the proper performance of their function. If it be unattended to, a greater share of work than ought to be is thrown upon certain of the internal organs—notably, the kidneys—from which disease is apt to arise. The proverbial saying that "cleanliness is next to godliness" would read better if it were put that "cleanliness is part of godliness." Dust and "dirt" are two things against which every cleanly housewife wages constant warfare. When the former is examined microscopically, it is found to consist of a large variety of particulate substances which are the products of the tear-and-wear of our bodies, and of objects around us. Dust always contains micro-organisms, and every dark, dusty corner harbours a large number. The prevalent mode of dry dusting is simply a farce, and consists only in dislocating the dust from the place on which it lies, into the atmosphere of the room, again to settle in a new place after the atmospheric disturbance has subsided. It is time that this method should give place to moist dusting by a damp cloth. It may mean more trouble, but it is worth it. Another old-fashioned method of sweeping a carpet, previously sprinkled over with moist spent tea leaves, is infinitely preferable to the more modern sweeping-made-easy machines of American origin. There is an old Scottish proverb, which must have descended from prehistoric times, which says, "The clartier (*i.e.*, the dirtier) the cosier." The followers of this precept have long since realised its falsity, for the Nemesis of insanitation has overtaken them. Fifty years ago, all over this kingdom, typhus fever raged as a plague. Even then it was pointed out that the plague raged worst in the dirtiest places. Now, owing to the forward march of sanitation, it is a comparatively rare disease.

A dirty person is usually a careless, a thriftless, and, very often, a vicious person, who is regardless of his responsibility to his relatives and to his neighbour. In a population composed of such persons sickness is more prevalent, and death-rates are always high.

Cleanliness in feeding is not only conducive to personal comfort, but it tends to prolong life. Good cooking of the humblest fare will nourish, while imperfect, uncleanly cooking of even the best will but provoke mischief. Flesh meat is probably the most common vehicle of parasitic disease. To avoid this, the Mosaic code laid down the strictest injunctions, and in the Talmud no fewer than 86 chapters, divided into 642 paragraphs, are devoted to this subject. So long as the present imperfect system of meat inspection obtains in this country, all flesh-meats should be thoroughly cooked, that is, till the fire has acted on every part of it. Thorough cooking renders parasitic germs innocuous. Cold pies of all sorts, brawn, corned beef, sausages, and "tinned" meats, have, at times, occasioned serious and, sometimes, fatal illnesses. Pies which are intended to be eaten cold should have two good-sized vent-holes in the crust. This lessens the chances of putrefaction. Micro-organisms, which find the "jelly" of the meat an excellent feeding-ground are prevented from thriving, by reason of the plentiful admission of air. Tinned meats only become dangerous when air has been admitted by a pin-hole opening some time prior to the time of using, and for the above reasons also. Care, therefore, should be exercised in the purchase of "canned" goods. A wholesome "can" should have its top and bottom tending inwards towards the centre of the "can," and should not emit a "crackling" sound when pressed by the finger. Any "can" which evidences the opposite of these characteristics ought to be rejected. These are, apparently, trifling facts, but they are very important, nevertheless.

Cleanliness of the Home.—The cleanly person has a cleanly home, and the converse is equally true. In addition, the dirty person and the dirty home menace, to some extent, the health and life of the neighbour. All the sanitary Acts are aimed against the careless and negligent, which terms are often synonymous with the dirty person. This is especially true of the spread of infectious disease from the home and school. Cleanliness of the home necessarily includes particular attention to the bath-room and water-closet.

Vicious Habits not only operate prejudicially on the health of the individual practisers, but, in the mass, they produce fruits in high mortality of offspring, in shortened lives generally, and in high death-rates. If we were to particularise regarding these habits, which are most harmful, we should name (1) abuse of alcohol; (2) of tobacco; (3) of tea; (4) immorality, as among the chief. Alcohol, in some form, has been manufactured by every nation of the earth, and from aboriginal times.

Occupational Habits.—It may fairly be said that there is hardly any occupation which is unattended by some objectionable habit on the part of those employed. Such habits may be divided into two classes, viz.: (1) those which are postural or attitudinal, owing to the position assumed by the worker, and which, too, are difficult to avoid; and (2) those which are only incidental to the occupation, and which might be avoided. Of the former class, are those where the employment compels the worker to assume a cramped or constrained attitude. Clerks, hand-loom weavers, authors, glovers, lacemakers, and shoemakers are instances; the stooping posture of the clerk or author at his desk, the pressure on the chest of the weaver or the shoemaker, by the loom and the "last" respectively, sooner or later provoke illness. Of the latter class are enervating occupations, such as are carried on in chemical or iron works, where heat is intense, or in badly-ventilated workshops, which invite the use of alcoholic stimulants and cause habits of intemperance. This is especially true of persons engaged in the manufacture, transport, or sale of alcohol. The mere negligence to clean the hands in such occupations as plumbing, type-setting, glazing, pottery-dipping, &c., accounts for much lead-poisoning.

Heredity, and its Influence on Health.—Each of us is the product of his or her ancestors. Each has had handed down as the legacy of his birth some of the physical, mental, and moral characteristics not only of the parents, but also of the grandparents, and of the ancestral line backwards. Regarding the transmission of the two former characteristics all are agreed. That moral defects are also hereditary, although doubted by some, is also now generally acknowledged. The children of depraved parents, who themselves being of similar stock, are likely to betray tendencies to kindred depravities; hence our dipsomaniacs from a drunken stock, and our moral delinquents from a theftuous ancestry. It is a difficult question, however, to determine in such cases how much of the delinquency is due to example, and how much to transmitted tendency. When we come to the possibility of transmission of disease from parent to child, it will be seen how hereditary conditions may affect the health of a population. In most of the diseases commonly believed to be transmissible, it ought to be remembered that it is not always the disease itself, but the tendency or vulnerability which is transmitted. All that is transmitted is the predisposition or cell-impression, and this makes the individual more liable to contract the disease when exposed to it under certain conditions. For example, the child of gouty ancestors may avoid the disease by a careful and constant attention to diet and regimen. Intermarriage with kindred blood, with and between weakly persons, or even diseased persons, is the cause of much transmitted tendency to disease. The marriage laws of the Australian aborigines, or of the American Indians, might well be copied by the civilised peoples of to-day, as they entirely prevented such contingencies. The physical aspects of marriage have not received the consideration they deserve, and if but a tithe of the care expended in the breeding of healthy stock were shown in our marriage relations, there would be much less disease resulting. State control can have no part or lot, however, in the attainment of this. Education only against the attendant evils of improper marriages may affect a change, but such will be achieved but slowly, since so many other considerations enter into the choice of a life-partner.

### Trained Nurses' Clinic.

### X.—THE NURSING OF OPERATION CASES.

Patients who have to undergo operations require very special care and attention from the trained nurse. To her the ordeal which they are about to face may sometimes appear a trivial one, but it is seldom regarded in that light by the patient. As a rule, the mere word operation causes a certain distressful excitement to arise in the minds of persons to whom "the unknown" presents hideous possibilities. Even the anæsthetic, which is part of the daily routine in a hospital nurse's experience, is often a dreary nightmare to the patient. Unaware of the thousands of people who have taken it safely, they remember with horror some newspaper report of one fatal case, which assumes disproportionate importance in this personal crisis.

The patient's over sensitiveness and perhaps irritability may be condoned, but they are not always understood, and he is conscious of appearing somewhat ridiculous to those around, whilst they remain ignorant of the real suffering which a quick imagination or even mere ignorance can bring upon a person of a nervous temperament. It requires the exercise of an equal amount of imagination to properly estimate the horrors of anticipation which a nervous person may live through on the eve of an operation, and it is probable that this is more often the case on small occasions than on great ones.

When much suffering is present, and tragic circumstances have tended to bring about the decision that operative interference is necessary, the patient may be so absorbed in his present condition as to be indifferent to the future trial.

On the contrary, the comparatively small matter being far less engrossing to the patient, his thoughts are free to wander most undesirably ahead. This in itself indicates what useful influence lies in the hands of a nurse.

Whatever amount of reparation she may have to make on the eve of an operation should of course take place without, so far as possible, the knowledge of the patient. Some nurses display infinite tact and consideration in this matter, whilst others are so little careful in their preparations as to greatly add to the alarm of the patient.

It is so hard to look at things with the eyes of other people that it may be thought unreasonable to expect a nurse of long experience to imagine herself in the position of a person who is scared by the sight of a bottle of chloroform and a basin and towel. Consideration and sympathy cannot be acquired in a day, therefore they should form from the first a part of a nurse's permanent equipment. She should never cease to respect the fears as well as the wishes of the sick, and the fact that both may be unreasonable does not acquit her of her obligations. To serve the patient without dictating to him can only be consistently accomplished by one who feels with as well as for her charges.

Whilst the operation itself depends on the surgeon, and for it he alone is held to be responsible, the nurse is of paramount importance in the hour before it takes place. To her the patient instinctively turns, auguring well from her quiet cheerfulness, whilst apt to be unreasonably depressed if her demeanour appear to him to be unduly solemn. The patient can hardly be expected to realise that she is influenced by interests other than his own. For the time being she is his nurse, and with not unnatural egotism he imagines her to be entirely engrossed with the consideration of his own condition.

The calm, reliable aspect of the competent and self-respecting nurse has an enormous influence on patients who are about to go through operations. They take such comfort from being with someone who knows "all about it." This is especially the case in a private house, which the nurse, on the eve of the operation, finds disorganised by the prospect of the event of the morrow. She is the one to whom all turn for advice, sympathy, and information, and she is naturally more lavish of the first than of the last. Whatever her knowledge and experience may have been she is generally wise enough to withhold both, and to make he icn versation encouraging without stimulating the appetite for sensationalism, which is by no means an uncommon accompaniment of personal excitement.

Of the preparation of room, patient, and accessories the trained nurse has full knowledge, but she needs also the intuition, which no amount of instruction can ensure, to make her all that is desirable both before and after the actual operation.

"I don't think I could have lived through the operation if it hadn't been for my nurse," is the verdict of many a nervous woman, and the heartfelt gratitude and relief exhibited, when all is happily over, may surely be accounted a sufficient reward by the woman who has felt with and for her troubled sister at a time when sympathy and consideration have a value not easy to estimate.

### Nurses in 1896—Their Quarters, Hours, and Food.

### MIDDLESEX HOSPITAL.

### I.—Terms of Training.

At the Middlesex Hospital probationer nurses are taken for training and are, if suitable, appointed staff nurses after one year in the hospital. For the first year they receive a salary of £12, being paid as staff nurses £18, rising £2 annually to £24. Sisters' salaries vary between £30 and £40 per annum. Lady probationers, of whom there are fifteen, pay £1 1s. weekly for six or twelve months' training. A gratuity of £1 per annum after five years' satisfactory service, and £2 per annum after ten years, is given to the sisters. A pension amounting to two-thirds of their wages is given to any of the staff after twenty years' service, while after fifteen years, if disabled by accident or sickness, a pension of not less than one-half the wages is allowed. Nurses receive a gratuity of £1 per annum after seven years' service, £2 after twelve. This Pension Fund is provided for from the lady probationers' fees, which have for years been funded for this purpose.

Certificates are not given at the end of any definite period, but on leaving the hospital. The non-paying probationers are almost wholly taken from among domestic servants, ladies not being admissible as candidates in that capacity. There is no prohibition against a nurse probationer becoming a Sister, though these appointments are usually filled from among the lady probationers. Examinations, as already mentioned, do not play the important part at Middlesex that they do at some other hospitals, very much more weight being attached by Miss Thorold to the practical knowledge of her work shown by a nurse in the wards. Lectures are, however, given regularly to the nurses, which they are required to attend during a part of the year, by members of the medical staff.

### II.—Hours of Work.

The hours on duty at Middlesex Hospital are very long, appallingly so having regard to what is required of the modern nurse in the course of her daily work in the wards. Probationers and staff nurses are on duty from 7.30 a.m. to 9 p.m., during which time they are off duty only on three days in the week, lectures also coming in off-duty hours. Lady probationers have a better time, being on duty for eight hours each day, from 8.30 a.m. to 8 p.m., and having three hours off duty each day. Sisters are on duty from 8.30 a.m. to 11 p.m., with two hours off daily, "five days weekly." Night nurses are on duty from 8.45 p.m. to 9 a.m. Seeing that attendance at lectures is expected from the probationers during nine months in the year in off duty time, the working day is really longer even than stated, and for at least four days in the week nurses are on duty, with the exception of meal-times, for thirteen and a half hours. Day and night work is taken in alternate months by the staff nurses; probationers are only given occasional night duty during their first year.

### III.—Holidays and Times off Duty.

Off-duty hours at Middlesex Hospital evidently date back in arrangement to the time when nurses, like servants, had "evenings out," for the week-day leave of absence is only from 5.45 p.m. On Sundays three hours are given, alternately in the morning, afternoon, and evening. Probationer nurses are allowed late passes till 11.20 once a month, and have one day off during their first year. Lady probationers are not given days off. Staff nurses have late passes till 11.20 once a month, and one day off duty alternate months. Sisters are off duty five days weekly, from 6 to 8.30 p.m., are permitted late passes till 11.30 twice a month, and have one day's leave monthly. Nurses on night duty are free every day from 10 a.m. to noon, or from 5.30 p.m. to 7.30 p.m.; Sundays, 10 to 1 p.m., or 5.30 p.m. to 8.30 p.m. No nurses are allowed to sleep away from the hospital on their days off-duty. Annual holidays are, for probationers, sixteen days at the end of year's training; for staff nurses, sixteen days annually; for sisters, three weeks annually. Except on Sundays it will be seen that none of the staff can get out into fresh air and exercise until so late in the afternoon, that for most months in the year it must be nearly, if not quite, dark, a practice which must be, in the end, very bad for the health of the nurse.

There are in actual fact certain modifications of the long hours given above, which possibly makes the work press less heavily than might at first be imagined. Miss Thorold's rule is a kindly one, and she gives much individual attention to the nurses, sending those who may be a little out of health away for a few days now and again, or giving a day off where specially asked for. It must also be said that the nurses at Middlesex are not required to pass examinations, which, whatever may be said of it from the training point of view, makes the actual work less of a strain.

### IV.—Uniform.

Nurses are provided with four dresses each during the year, two print and two of Russell cord. Fourteen aprons are given at first and renewed as required, and four caps yearly. The sisters wear violet serge, and have two dresses of this material given yearly, with caps and aprons. The nurses' caps and aprons are washed in the hospital laundry; no other allowance is made.

### V.—Nurses' Quarters.

The nurses' home at the Middlesex Hospital is immediately attached to the hospital proper, shut off by a door of communication. Here all the nurses working in the hospital are lodged, with the exception of the sisters of the wards, who have bed-sitting-rooms outside their wards. The home is separate to, but adjoining the Private Nursing Institute, which has its own entrance in Cleveland Street, and some of the rooms in the institute are useful by way of overflow accommodation for the hospital nurses, for the home is now not large enough for the staff, and when the new cancer wards are erected an enlargement of the nurses' quarters will be imperative. There are separate bed-rooms and cubicles for the nurses, and two sitting-rooms, one for the nurses and one for the lady probationers, comfortable rooms, and nicely furnished. The dining-room is rather a dismal apartment in the basement, close to the kitchen, and low and dark, for some of the windows are faced by the wall of a part of the hospital building. Here are a number of tables, one of which is reserved for the sisters, and the meals are all presided over by the "housekeeper," who is in charge of the home. There is a separate kitchen, which has been recently fitted with an excellent gas cooking apparatus. One room was originally set aside as a sick-room for the nursing staff, but owing to the limited space it has been taken into general use. Nurses, therefore, who are ill (sickness amongst the Middlesex nurses is said to be of rare occurrence) are treated in their own rooms or in the wards. The sisters' bed-sitting-rooms are quite outside the wards at Middlesex, and are pleasant and cheerful.

### VI.—Meals.

The nurses' meals are presided over by a home sister or housekeeper, and are all served in the common dining-hall in the home. The dietary it liberal, and varied as far a possible. For breakfast the nurses have tea and coffee, fried bacon, sausages, fish, potted meats, jam, and marmalade. At dinner, meat, two vegetables, puddings, fruit tarts, and beer, lemonade, and soda water. The day nurses' dinners are at wonderfully early hours at Middlesex Hospital, *i.e.*, 10.45 and 11.30 a.m. An early lunch is thus dispensed with, and three-quarters of an hour is allowed for dinner. Tea is laid for the nurses in the dining-room at 4 o'clock and 4.30; and supper, consisting of meat and cheese, with bread and butter, coffee, beer, or milk is served at 9 for the staff nurses and nurse probationers, the lady probationers having theirs when they come off duty at 8. In the night nurses' ward the meals consist of sardines, ham, potted meat, cake, eggs, jam, and marmalade, with coffee, and bread and butter, and these provisions are taken from the kitchen every night, and the meal taken in the ward kitchen. Fish is provided on Fridays for those who wish it. There is no limit placed on the food supply at the Middlesex Hospital.

### A South African Hospital.

GRAHAMSTOWN is one of the prettiest towns—if not the prettiest town—in all the Cape Colony. The situation is lovely, lying as it does in a verdant valley, watered by two rivers, and surrounded on every side with high grass-covered hills. Moreover, the wisdom of Government has ordained that on these hills a certain number of trees shall be planted every year, so that, within the last thirty or forty years, groves of pine, gum, and other evergreen trees have sprung up on every hill, clothing them with perennial verdure and beauty. The town itself consists of very long, broad streets, running at right angles to each other, up hill and down hill, and planted on either side with rows of trees, some of which have grown to a considerable height. On West Hill, which may be called a suburb of the town, are the houses of the well-to-do, surrounded with more trees, and standing in lovely gardens, brilliant with flowers. There are more churches and schools in Grahamstown than in almost any other town of the size; but I am writing too much of the town, instead of confining myself to the subject of my paper—the hospital.

The hospital at Grahamstown—called the Albany General Hospital, inasmuch as Grahamstown is in the district of Albany—is a good match for the town, in that a prettier hospital, in a prettier situation, could not well be found. It is not large; it accommodates 80 patients; and is nearly always full. It stands a little way out of the town, on the side of the hill, high enough to command an extensive view of the town and the surrounding country. The trees about it are near enough to shut it in to a certain extent, but not so near as to make it dark or unwholesome Indeed one could not wish for a brighter, more cheerful-looking building, inside and out. It is rather long and narrow in shape, with a short staircase at either end, being a two-storey building. The wards are small, of course, and perhaps rather bare-looking to English eyes—superfluous draperies do not find much favour in this land of dust and burning sunshine — yet they are thoroughly homelike and cheerful, and it is unnecessary to say that cleanliness and order prevail in every part. There are, of course, as in all colonial institutions, separate wards for the white and for the coloured population, and the hospital therefore contains two wards for men, two for women, and a children's ward for white children only. There is an operating room, in one corner of which is a dark closet, arranged for the operations of the oculist; and at either end of the building are two or three rooms for private patients, the men at one end, the women at the other. Everything, on a small scale, is very complete.

I have not yet spoken of the chief beauty of the hospital—its verandahs, or, as we call them here, stoeps. They run the whole length of the building, both back and front, upstairs and downstairs. They have boarded floors, are very broad, and well shaded with luxuriant creepers; there are seats and chairs of all sorts on them. A more delightful resort for convalescent patients cannot be imagined. When we went through the hospital, we saw so many empty beds, that I began to think the place was only half full; but I soon discovered that every patient who was able to be up was out on the stoep, enjoying the balmy air and the lovely view. At either end of the upper stoep, opposite the private rooms, a portion is divided off with a wooden partition for the use of the private patients.

There are three separate buildings attached to the hospital—a nurses' home, the doctor's house, and the isolation wards for infectious cases. I did not see the inside of any of these buildings—the two first-named are quite new—but inside the hospital itself I saw a nice comfortable-looking nurses' dining room, a small—too small—sitting-room for the matron, and one of the nurse's bedrooms, also small, of course, and plain, but comfortable, and of a good height, which is, after all, the great consideration in a bedroom. There is also a library for the nurses, stowed away in the corner, containing, perhaps, 200 volumes, and very inviting in aspect.

In this, as in all colonial hospitals, nurses have the great advantage of learning to do all the work that is done by the medical students at home. The medical student is unknown in the colony; all colonial doctors have studied and passed their examinations in the old country; and the consequence is that dressings, &c., are done by the nurses, who thus acquire an experience which they have not the opportunity of acquiring in an English hospital.

There is a good piece of ground in front of the Albany Hospital, which has been nicely planted out with trees and shrubs, and in front of the nurses' home is a lawn tennis court. One recent improvement in the hospital and everywhere else is the introduction of gas, which has only lately come to illuminate the darkness of Grahamstown. Now we wonder how we existed in the days—or rather nights—of paraffin lamps and lanterns. Many a night, when on duty at the asylum, have I gone out on the stoep and looked across the broad valley at the hospital on the opposite hill, about a mile away, and watched the solitary twinkling lights, and felt a tacit companionship with the night nurse, who, like myself, was going her darkling rounds. "Nous avons changé tout celà," and the Grahamstown Asylum and the Albany Hospital have become bright and shining lights, moral and material beacons to the surrounding country.

### Royal British Nurses' Association.

SEVERAL of the members of the Royal British Nurses' Association who were present at the annual meeting have called our attention to the statement in a contemporary that "there was a large attendance of members, the nurses from the Middlesex Hospital and the Chelsea Workhouse Infirmary being especially noticeable in large numbers." Our correspondents stated that they are well acquainted with the nurses of the staff of the hospitals in question, that the statement referred to is incorrect in every particular, and that, as a matter of fact, not more than four nurses from the Chelsea Workhouse Infirmary were present at the annual meeting on the 25th ult.

### Novelties for Nurses.

### NURSING SPECIALITIES.

MESSRS. REYNOLDS AND BRANSON, of Leeds, are issuing a catalogue of their specialities, which will be found very useful to nurses for handy reference. We have frequently noticed the unique and useful inventions which emanate from this firm from time to time, and recommend them to all interested in appliances which add to the convenience of nursing and the comfort of the sick.

### Holidays and Health.

[Readers of The Hospital in need of information about health resorts at home or abroad, or desirous of aid in forming holiday plans, are invited to send queries to Editor, 428, Strand, W.C. (marked "Travel" on outside of envelope), which will be answered under this section.]

### LONDON TO KILLARNEY.

The charms of the Emerald Isle, and the advantages it offers to holiday seekers on the one hand, and the alleged discomforts and disadvantages which beset the unwary visitor on the other, have been lately much *en evidence*; the theme of discussion by an admiring association wishing to attract, and the cause of much adverse criticism by numerous correspondents in the *Standard*.

It is, perhaps, possible, like the landlord of the roadside public house, in George Eliot's inimitable "Silas Marner," to agree with both admirers and detractors, and yet so adjust the balance as to get the maximum of Ould Ireland's beauties and advantages with a minimum of its disadvantages. The complaints lately made in no way depreciate the charm of the scenery, as fresh and beautiful as ever, but are directed against the accommodation afforded to tourists by the railway and hotels, and it may be possible to point out a route by which these serious obstacles to the enjoyment of a real holiday can be avoided.

Intending holiday makers may like to hear of one of the most delightful expeditions to the Isle of St. Patrick, taken by the writer some years ago, of which the pleasant memory yet abides.

Now it will be found that a nameless charm is lent to a holiday, if some portion, however small, of the "silver streak" is interposed between one's place of work and the land of holiday and rest, and this is vastly increased if you can, in the first place, elude the hurry and scramble of a railway terminus and make a start by the more peaceful water. Moreover, there is no rest so altogether useful as the isolation, if only for three days, afforded by a boat, away from letters, telegrams, and the eternal post. A start is, therefore, suggested by one of the steamers of the City of Cork Steam Packet Company from the London Docks, which run from there to Cork, three days steaming, with two or, if preferred, three nights on board.

Given a fine morning in July or August there are no bounds to the pleasurable feeling of putting some miles of water between work and holiday. Going by a boat which starts quite early in the morning you can, if you please, go on board overnight without extra charge, and though it, probably, won't be much of a night, you are quite disposed to imagine you have had a most refreshing sleep, and to enjoy the unaccustomed sights and sounds. There is a positive delight in embarking in a Thames wherry, with a real Thames waterman, going a little way down the river by moonlight or starlight, and then back again in time for "lights out" on board the steamer, giving oneself up to the stirring and novel scenes on all sides. Below "bridge"; *the* bridge, *par excellence*, of London city. Think of it, as it was, the only bridge for centuries that spanned silver Thames, with its quaint houses, its beautiful chapel, and, alas! its ghastly memories.

Then the glimpse of the frowning fortress, the Tower of London, with the Traitor's Gate, which no one can pass without a shudder, through which so many more sinned against than sinning have passed to their doom, and the great white Norman tower, recalling the scenes on which it has looked, and marvelling that it was not long ago razed to the ground by a people who have since so successfully "rightly struggled to be free." Then passing under the noble Tower Bridge, meet river gate of London, then the Pool, threading a devious way through shipping from all parts of the world, and so down the widening river, past Greenwich, its Park, Observatory, and Hospital, where one still misses the old pensioners; and so into the open sea.

The steamer keeps well within sight of the coast, and very pretty it is skirting Essex, Kent, Sussex, Hampshire, with the cliffs of the Isle of Wight, past Portland and the Dorset coast, and then by sunny Devon, reaching the beautiful harbour of Plymouth, if you are lucky, somewhere about sunset, the coast and the red sandstone rocks, whose half-day's bath is done, roseate in colours and altogether lovely.

Plymouth Sound, too, is full of memories of Devon's worthy sons who sailed from thence to meet the Spanish invaders threatening to overwhelm the land of "Gloriana." There, too, is the place where Drake and his friends played and finished their wondrous game of bowls almost in sight of the enemy, strong in their righteous cause and in loyalty to their Queen and country.

Charter a boat over night, make a very early start, row across the harbour, and land in Mount Edgecombe's beautiful park, and then back to the steamer for breakfast for a fresh start. In sight are the charming Devon and Cornish shores, Falmouth Bay, and the Land's End, with, if it be clear, just a peep at Scilly, and so across the open sea to the mouth of Cork river.

The steam up the river, which is very pretty, is very enjoyable, and you find yourself greatly refreshed and very hungry, on *terrâ firma* once more, in Ould Ireland. A run through Cork, and a visit to Blarney Castle and one or two places of interest should be made, or else start direct by the Cork and Blandon Railway to Bantry. Here a coach or car meets the train, and a charming drive along the coast, with Bantry Bay on the left, brings you in eleven or twelve miles to Glengariff.

It is difficult to describe the beauties of Glengariff, there is really nothing like it anywhere else. Beautifully wooded right down to the Salt Water Lake, which is studded with green islets, it is indeed ideal, and as the hotels are excellent and the charges reasonable, and pleasant people abound, while there is some charming scenery to be seen and revelled in, it is advisable to spend at least some days in this lovely spot. From Glengariff by coach to Kenmare, and if you are ready for an enjoyable walk through the mountains, it is best here to leave the coach, sending on your luggage, and walk to Killarney.

The view from the top of the hill overlooking the lakes, of which here is obtained the first glimpse, is magnificent. If you have taken Cook's tickets you will of course go to the hotel where they are available. If not, you cannot do better than stay at the Muckross Hotel, about a mile from Killarney itself, close to the Abbey and the lakes, which is very comfortable and convenient. All information as to tickets, hotel coupons, &c., which saves no end of trouble, and money, can be obtained from Messrs. Cook's "Guide" to the Emerald Isle.

From Killarney the ascent should be made of the McGillicuddy Reeks, which will be found quite easy after a few days' good walking to get into training. On any walking expeditions it is a good rule to remember to "take the first mile easy." Inexperienced and enthusiastic pedestrians often make the great mistake of beginning too fast. Strong boots, with football bars or nails are necessary; above all things the boots should not be new. Another great comfort on such a holiday is a small india-rubber bath, for use on the steamer, particularly, but it will also be found very useful afterwards. These may seem trivial details, but such small matters sometimes make or mar the holiday.

There are really few places in the world more beautiful than Killarney. It is impossible to tire of those lovely lakes, and each day you will discover new beauties; but one cannot, in a sketch of this kind, do more than generalise. It will be found far better not to attempt too much sight-seeing, but be content, spend the rest of your time here until the fateful hour when steps must be turned homeward again. There is enough in this unpretending little tour to store the mind with most pleasant memories, and to brace the nerves up for another year's work. The expense of it is trifling. It is no doubt possible to arrange for the return journey to be varied by steaming only from Cork to Bristol, and thence by Great Western Railway to Paddington, or direct to London by the same route you came.

A return ticket, available for two months, from London to Cork is only 30s., cabin fare, and Messrs. Cook issue both railway and hotel coupons, which provide the best accommodation, at a most reasonable rate, including conveyances to and from the different places of interest, boats, and boatmen on the lake.

### A Book and its Story.

### "THE MIGHTY ATOM."

This is a novel* with a purpose—to protest against excluding the teaching of religion from the training of children, and against the evils of over-education. But the novel with a purpose is a weapon which can never be effective, in any controversy, unless it is used with discretion, and without passion.

Now, among those who desire a system of purely secular education there are thousands who believe implicitely in the doctrines of the Christian faith, who instruct their own children in that faith, and whose domestic lives are examples of all the virtues which it is capable of producing.

Of this fact Miss Corelli must be perfectly aware, and yet she has prefixed to the volume before us a dedication, in which (after assuming apparently that all the Progressive party—to use the common phrase—are Secularists) she makes some striking assertions, amongst others "that the Progressivists," by precept and example, are supporting an "infamous cause," &c. It is not by virulent and abusive language, or by insulting our opponents, that those of us who object to the separation of religion from education can hope to secure the victory of what we hold to be right. The authoress of "The Mighty Atom" has sometimes shown herself morbidly sensitive on the subject of hostile criticism, and we fear by certain utterances in her latest contribution to literature she will lay herself open to attacks which she may have some difficulty in repelling.

Miss Corelli writes nothing which is not worth reading, and this story, though not rising to so high a level as "Barabbas" and "The Sorrows of Satan," is a fitting successor to those able works. The prevailing note is one of sadness. John Valliscourt, of Valliscourt, is a learned man, an atheist, or at least an agnostic. His wife is a woman of the world, and a beauty. "Her smile was a beautiful one—her large flashing eyes and brilliantly white teeth gave it a sun-like dazzle that amazed and half bewitched any man who was not quite prepared to meet it." They have an only son, Lionel, whose brain has been overworked from infancy. He is a clever, and extremely precocious little man, just ten years old. Young as he is, he has already a considerable knowledge of Latin, Greek, and history. "His store of purely scientific information was great." He was far advanced in mathematics. "The boy's bright brain pounced, as it were, on a difficult proposition of Euclid, and solved it without difficulty." And this was the result of over-pressure, and had been brought about at the cost of great suffering. As to religion, "he had been duly instructed in the form of the Christian 'myth,' as a myth only, in company with all the other creeds known to history. He had been carefully taught that only barbarians believed nowadays in the supernatural. The intellectual classes fully understood—so he was told—that there was no God, and that the first cause of the universe was merely an atom, productive of other atoms, which moved in circles of fortuitous regularity, shaping worlds indifferently, and without any mind-force whatever behind the visible matter."

Whilst he is studying at home with his tutor, Professor Cadman-Gore, the young life of Lionel is overshadowed by a terrible grief.

A certain Sir Charles Lascelles, "a languid, handsome-featured person," is on intimate terms with Mr. and Mrs. Valliscourt. Mrs. Valliscourt is bored to death by her husband, and Sir Charles, who is a frequent visitor, does not hesitate to take advantage of the position. At last, one evening, when Mr. Valliscourt and Professor Cadman are, by chance, absent on an excursion, Lionel is wakened by his mother, who tells him that she is going away. The boy has a dim intuition that something dreadful is about to happen. "Mother, don't go!" he pleaded, "stay to-night at any rate! Wait till to-morrow—oh, do, mother! Don't leave me!" But he pleads in vain. Her mind is made up; she is about to run off with Sir Charles.

"Oh, you don't know," she exclaims, "how dull respectability can be! how insufferably, hopelessly dull! You don't know—you can't understand—that when the object in life is to be respectable and nothing more—no other ambition, no other future, no other end—it becomes deadly!—even desperate."

Shortly after these words Lionel's mother elopes, leaving a note for her husband, who returns next morning with the professor, and pours forth a torrent of invective against his wife when he discovers what has taken place. "When you are a man," he shouts to Lionel, "you will blush to think she ever was your mother. . . . She has made herself a scandal to society. She is a debased and degraded example of impudence, dishonesty, and infamy! She—" But here Lionel stumbled forward giddily, and laid his weak little hand appealingly on his father's arm. "Oh no! father, no! I can't bear it," he cried. "I love her! I love her!" It is soon evident that the shock has been too much for the boy's feeble body and overtasked brain. A serious illness follows. From this he recovers, only, however, to find that a little village girl with whom he had stolen, from time to time, a few hours of play (there is a very sweet and charming picture of their childish friendship) is dead. The boy is overwhelmed with sorrow, and his is a sorrow without hope. When her father speaks of God, and faith, and a meeting beyond the grave, Lionel sobs "No, no! there is no God! You have not read, you have not studied things, and you do not know; but you are all wrong! There is no God—there is only an Atom, which does not care."

From this point the tragedy hurries to its close. "Perhaps," Lionel says to himself, "the learned men are not as wise as they think; it is possible they may be mistaken. The Atom they argue about may be a God after all . . . and there may be another life after this one, and another world, where Jessamine is now. The question is, how to be quite, quite sure of it." The boy ponders for some time on this problem of the future life, and at last a sudden thought flashes across his mind. "I know," he whispers, "I know the best way to discover the real secret—I must find it out; and I will!" And accordingly one night he makes up his mind to take the only sure and certain way to solve the great question. "Then he went to the window and opened it. It was a glorious night, and as he threw back the lattice a sweet air flowed in, laden with a thousand delicious odours from the forest and ocean." Then he prays to the Mighty Atom, which is for him the Great First Cause, and passes away into the unknown. "And not another sound disturbed the quietude of the house, save the quick, dull 'thud' of a chair overturned and thrown down. After that came a heavy stillness, and a sudden sense of cold in the air, as of the swift passing of the Shadow of Death."

In the morning the poor little boy is found dead, hanging from the ceiling of the room, the victim of the false system under which his father had educated him. Such in brief outline is the story of "The Mighty Atom." It abounds in pathos, and contains much that is suggestive and much that is true. But we must repeat our regret that the brilliant writer should, in support of a good cause, have been betrayed into expressions which are calculated to hinder rather than to advance the interests of religious education.

* "The Mighty Atom," Marie Corelli. London, 1896.

### Ethics of Nursing.

(*Continued from page* clii.)

THE DUTY OF THE NURSE TO THE PATIENT.

SEC. 1.—Except on substantial grounds, a nurse should never refuse a call to a sick person. She should never allow her personal inclination or her personal pleasure to interfere with this duty. It is equally binding upon her as upon a physician to go when summoned.

SEC. 2.—She should be deeply conscious of the grave responsibility of her position, and in no case should she be guilty of carelessness or neglect of any duty that skill, attention, and fidelity upon her part should bestow.

SEC. 3.—Every patient committed to the care of a nurse should be treated with attention, steadiness, and humanity. Although proper firmness is necessary, it should never be allowed to degenerate into severity, and reasonable indulgence should be granted to the caprices of the sick, more especially to those whose mental powers are affected. Too great intimacy between the patient and nurse is not to be encouraged, but the confidential intercourse to which nurses are admitted should be used with the utmost discretion and with the most scrupulous regard to fidelity and honour. The obligation of secrecy extends beyond the period of professional services; none of the privacies of personal and domestic life, no infirmity of disposition or flaw of character, observed during professional services, should ever be divulged by the nurse unless circumstances arise which render such a course an imperative duty. The same rule holds good also with respect to the patient's ailments. Patients and their affairs should not be made a subject for conversation or discussion between nurses; silence is even more binding upon the nurse than upon the physician, as the opportunities of the former for knowing her patient's affairs are often greater than those of the latter.

SEC. 4.—A nurse should not leave a patient on account of her own private affairs or because her position is rendered disagreeable to her through the friends or the physician, unless she is assured that her presence is displeasing to the patient. When it is absolutely necessary for her to leave she should be willing to remain until some competent nurse has been found to take her place.

SEC. 5.—Engagements, whether written or verbal, should always be regarded as legal contracts. The rescinding by the nurse of such a contract is only justifiable in the face of unavoidable emergencies, or when the maintenance of her self-respect is clearly involved.

### The Mary Wardell Convalescent Home.

It is now eleven years since Miss Mary Wardell, led by her experience in working amongst the London poor to feel the urgent need for some place to which sufferers from scarlet fever could be sent for change of air, established, with the warm encouragement of the late Sir Andrew Clark and many medical men, the first convalescent home for scarlet fever cases at Stanmore, Brockley Hill. Since then more than 2,400 patients have been admitted to the home, which has thus conferred an incalculable benefit on the public by providing a refuge for those who have gone through this highly infectious fever, and need for themselves fresh air and exercise, under careful supervision, whilst still a source of danger to their neighbours.

Though originally intended for the benefit of the poorer classes, in consequence of special requests, accommodation has been provided for people who are able to pay. These payments, however, do not cover any part of the expenditure on the charitable branch of the institution, and funds are now sorely needed to meet the yearly expenses and to pay off a debt incurred in the repainting, whitewashing, and disinfecting of the home, necessary after several years' work. For various obvious reasons the home is more expensive in its working than an ordinary convalescent home. A special omnibus has to be kept for the transport of patients from hospital or their own homes, necessitating a horse and man. Then the irregularity of the demand for admission, exceptionally heavy at one time, whilst at another the staff and housekeeping arrangements needful for these seasons of pressure are in excess of requirements, is a difficulty, particularly as outside assistance is impossible to obtain on short notice. The inmates are usually more numerous in the winter months, when domestic expenditure is highest, with firing and lighting; and lastly, the disinfecting arrangements and laundry are expensive items in the year's accounts. The house stands on the top of Brockley Hill, 450 feet above the sea level, on gravel soil, with a southern and western aspect. It stands on a freehold of four acres, laid out partly in kitchen gardens, partly as a pleasure ground, where there are tennis and croquet lawns for the convalescents. The house is planned for the accommodation of about forty patients. At present only women and children (boys under 12) are admitted, funds being greatly wanted to add the necessary rooms for men, and provide further facilities for isolation. During 1895, 247 patients were admitted, of whom 79 had been previously treated at their own homes, the rest at various other hospitals. A visiting medical officer attends the home twice a week, and besides the matron, Miss Cahusac, there is a trained head-nurse. Admission is by payment, patients in the first-class department paying three guineas a week; patients from the working classes, fifteen shillings; and children under twelve, ten shillings a week. The home may well claim to be a friend to all other institutions, being a means of security against the spread of infection, as well as affording a most real boon to the sufferers themselves. Subscriptions and donations are earnestly asked for, and will be received by Messrs. Barclay, Bevan, Ransom, and Co., 54, Lombard Street, E.C., and by Miss Mary Wardell, Hon. Secretary, Convalescent Home, Stanmore, Middlesex, from whom all information, papers for circulation, collecting cards, &c., can be obtained.

### Appointments.

### MATRONS.

RAMSAY ISOLATION HOSPITAL, ISLE OF MAN.—Miss Leila Murray has been appointed Matron to this hospital. She was trained at the Isle of Man General Hospital, Douglas, and at the Borough Sanatorium, Douglas, where she was occupied in fever nursing. She has since devoted herself to private nursing.

CLIFTON COLLEGE SANATORIUM.—Miss Ada Plumb has been appointed Matron of the above. She was trained at the Royal Infirmary, Edinburgh, for the Metropolitan and National Nursing Association, and held the posts of sister at the Hospital for Women, Soho Square, and matron of the Chelsea Hospital for Women.

HOME FOR TRAINED NURSES, BATH.—Miss F. E. Latham has been appointed Lady Superintendent of the Home for Trained Nurses, 44, Rivers Street, Bath. Miss Latham was trained at the Royal Infirmary, Bristol, and at the Rotunda Hospital, Dublin. She occupied the post of charge nurse at Bristol until 1895, and until her recent appointment was engaged in private nursing and as night superintendent at the East Dulwich Grove Infirmary. Miss Latham has most excellent testimonials.

BROOK FEVER HOSPITAI, SHOOTER'S HILL.—Miss E. M. Bann has been appointed Ma ron. She was trained at the Royal Infirmary, Derby, and afterwards occupied the position of charge nurse of the scarlet fever wards, and of the women and children's ward respectively. She later held the post of night superintendent at the General Infirmary, Gloucester, and since 1892 she has been lady superintendent of the Sanatorium, Hull. Miss Bann's testimonials are exceptionally excellent, and we wish her all success in her new and important post.

### Reading to the Sick.

GOD'S TEACHING.

**Motto.**

Be silent to God, and let Him mould thee.—*Ps.* xxxvii.

**Verses.**

Surely the time is short,
Endless the task and art,
To brighten for the ethereal court
A soil'd earth-drudging heart;
But He, the dread Proclaimer of that hour,
Is pledged to thee in love as to thy foes in power.
—*Keble.*

Unto you is given,
To watch for the coming of His feet,
Who is the glory of our blessed Heaven;
Even in an earthly home,
And in such an hour as you think not
He will come.
—*B. M.*

Can I teach thee, my beloved, can I teach thee?
If I said, "Go left!—or right!"
The counsel would be light,
The wisdom, poor of all that could enrich thee;
My Right would show thee Left.
My raising would depress thee,
My choice of light would blind thee—
Of way,—would leave behind thee,
Of end,—would leave bereft—
Alas! I can but bless thee!
May God teach thee, my belovèd! May God teach thee!
—*E. B. Browning.*

**Reading.**

"There is a true rest in resigning ourselves to be taught; in yielding ourselves to the leading of His Spirit; in coming to prayer and to worship, and to the daily duties of our station; not as if, through these things, we were to work ourselves up to great attainments, but as that course, in which He, for Christ's sake, will meet us."

—*Bishop Wilberforce.*

In the Ages of Faith, when it was believed that this or that shrine possessed a piece of the true Cross, men thought no labour too great, no journey too difficult, if only they might touch so sacred a relic. Their desire still lives in us; could we but feel that our Cross was a part of Christ's Cross, how gladly would we bear it! We have many crosses, but we are apt to lay them to the charge of that "unspiritual god—circumstances." We fret against our unsympathetic friends, or our parents; our poverty, which makes us dress badly, and give up more or less expensive pleasures; our stupidity, which brings ill-success, in spite of hard work; we pity ourselves for these annoyances, and never realize that therein lies our great chance in life—our chance of saintliness. Perhaps the cross of sickness is more easily seen in its true light—its true glory—than many others. Health and sickness are more visibly the direct appointment of God than, for instance, the happiness or misery caused by the temper of those around us. If you are weighed down by pain, which is "the thing that comes closest from God's hand," consider how many people, outwardly more prosperous, find their crosses harder to bear, because it is so difficult to refer them to their first cause. Their burden seems so made up of blunders and cross purposes, that they have none of your pleasurable feeling of enduring something specially assigned by God Himself. No matter whether it be our own fault that things have gone so crookedly, let us take things bravely and sweet-temperedly for the Master's sake, and then each anxiety or throb of pain will become—in spite of its inevitableness—a voluntary sharing of the Master's burden. —*L. H. M. Soulsby.*

### Everybody's Opinion.

[Correspondence on all subjects is invited, but we cannot in any way be responsible for the opinions expressed by our correspondents. No communications can be entertained if the name and address of the correspondent is not given, or unless one side of the paper only be written on.]

THE NON-EMPLOYMENT OF ATKINS.

Dr. James, treasurer of the Hamilton Association for Trained Male Nurses, writes: Referring to the interesting communication in your current number from "A Superintendent of the Indian Nursing Service," will you permit me to say that nurses trained in the manner stated are eligible for entry on the roll of the Hamilton Association for Providing Trained Male Nurses (57, Park Street, Grosvenor Square)? There are, in fact, vacancies at the present moment for first-class highly-trained men, and I can endorse the good opinion of your contributor respecting the qualities of army trained nurses, a number of whom have entered the Hamilton Association. Many of your readers are aware that this association was established more than a decade ago by the late Miss Hamilton, for the purpose of providing nurses for such cases among others as your contributor mentions, and also with a view to enable trained men to meet with the employment for which they had shown their aptitude. Miss Hamilton devoted large sums of money to this benevolent purpose, and the work is being carried on by a committee, on which the medical profession, military and civil, is strongly represented. The eleventh annual meeting is to be held to-day. The report to be presented shows a decreased deficiency, but still a deficiency of income, and it is hoped those interested will so far encourage the enterprise that the association will soon be self-supporting. You are no doubt aware that the Hamilton Association was the pioneer in this philanthropic work. Others have since professed to do a part of it. The committee make no objection to competition, but they deprecate the supply of any but highly-trained men. For these they desire to secure further employment, and they wish at the same time to press on the public the propriety of availing themselves of the assistance of men in numerous cases for which they are more fitted than women.

### Minor Appointments.

Cheshire County Asylum.—Miss L. Haslewood, who was elected head nurse at the above institution, has since withdrawn her application.

St. Thomas's Hospital.—Miss Florence Haig Brown has been appointed Home Sister at this hospital. Miss Haig Brown was trained at St. Thomas's Hospital, and afterwards held the post of Night Superintendent at the Brompton Hospital for Consumption. She at present fills the post of Home Sister and Assistant Matron at St. Marylebone Infirmary. She will take up her duties at the end of the month.

### Where to Go.

East London Exhibition.—Miss Gethen (formerly Sister Queen of the London Hospital) will relate some original stories or word pictures of hospital life at the People's Palace, Mile End Road, each Thursday in August at half-past five and seven o'clock.

In Aid of Guy's Hospital.—A garden party and fête will be held in Harrod's Depository Grounds, Barnes, on Saturday, August 8th, 1896. The employés of Harrod's Stores are holding this fête to commemorate the inauguration of Saturday Early Closing, which will commence on August 8th. They will devote the entire proceeds from the tickets to the funds of Guy's Hospital.

